# Assessment of the impact of cataract surgery on subjective quality of vision across different intraocular lens type using the portuguese-validated QoV questionnaire

**DOI:** 10.1007/s10792-026-04172-x

**Published:** 2026-07-20

**Authors:** Glendha Santos Pereira, Juliana Frange de Miranda, Konrad Pesudovs, Julia Mendonça Ponte Souza, Wilson Takashi Hida, Ricardo Yuji Abe

**Affiliations:** 1 Hospital Oftalmológico de Brasília, SGAS 607 Avenida L2 Sul, Brasília, Distrito Federal Brazil; 2 School of Optometry and Vision Science, University of New South Wales (UNSW), Sydney, Australia; 3 Department of Ophthalmology, University of Campinas, Campinas, Brazil

**Keywords:** Cataract surgery, Quality of vision, Intraocular lenses, Rasch analysis, Patient-reported outcomes

## Abstract

**Background:**

Investigating cataract surgery outcomes is essential to capture patients’ subjective perception of visual quality. This study evaluated quality of vision (QoV) before and after cataract surgery by comparing different intraocular lens (IOL) models using a Rasch-validated Brazilian Portuguese version of the QoV questionnaire.

**Methods:**

This prospective study recruited patients from the Cataract Department of the Hospital Oftalmológico de Brasília. Participants were ≥ 50 years old, underwent bilateral phacoemulsification with implantation of the same IOL model, and had preoperative best-corrected visual acuity of 20/50 or better. Included IOLs were monofocal, premium monofocal, extended depth of focus (EDOF), and multifocal lenses. The Rasch-validated QoV questionnaire was administered pre- and postoperatively.

**Results:**

Fifty-eight patients were analyzed: 10% received EDOF, 18% monofocal, and 70% trifocal IOLs (p < 0.001). Mean age was 64.53 years. For the whole cohort, median satisfaction with uncorrected distance vision increased from 7.50 preoperatively to 8.00 postoperatively (*p* = 0.002). Median near-vision satisfaction improved from 3.50 to 8.00 (*p* < 0.001). Median uncorrected visual acuity improved from 0.40 to 0.00 logMAR in both eyes (*p* < 0.001). In the monofocal group, QoV frequency scores decreased from 35.48 to 21.36 (*p* = 0.047) and severity scores from 33.87 to 16.99 (*p* = 0.019), indicating fewer symptoms. Although visual acuity and patient satisfaction significantly improved after surgery, no significant differences in QoV outcomes were identified between IOL groups in the linear mixed-effects models, which constituted the primary inferential analysis.

**Conclusions:**

Cataract surgery significantly improved visual acuity and patient-reported satisfaction. However, no statistically significant differences in QoV outcomes were detected between IOL categories during the early postoperative period. Given the small and highly unbalanced sample, these findings should not be interpreted as evidence of equivalence among IOL types, and the study may have been underpowered to detect subtle but clinically meaningful differences.

**Supplementary Information:**

The online version contains supplementary material available at 10.1007/s10792-026-04172-x.

## Introduction

Visual quality is a central factor for functionality and quality of life, especially among the elderly. With the aging of the population, cataract remains the leading cause of reversible visual loss worldwide [1, 2]. Despite significant advances in surgical techniques and intraocular lenses (IOLs), the assessment of cataract surgery outcomes should go beyond corrected visual acuity and include patients’ subjective perception [3, 4]. For this purpose, specific instruments have been developed to measure the subjective quality of vision [5, 6].

The Quality of Vision (QoV) questionnaire is widely used to assess symptoms such as halos, glare, and blurred vision, considering three dimensions: frequency, severity, and bothersome. Based on Rasch analysis, the QoV demonstrates high psychometric reliability and is widely used to compare different types of IOLs [7]. However, there is no validated Brazilian Portuguese version of the QoV, despite the existence of other instruments, such as the 25-Item National Eye Institute Visual Function Questionnaire (NEI VFQ-25), which do not detail specific visual symptoms for cataract surgery outcomes using different IOLs [8, 9].

Thus, this study aims to evaluate the subjective perception of QoV before and after cataract surgery by comparing different models of IOLs, using a validated Brazilian Portuguese translation of the QoV questionnaire.

## Methods

### Participants

This was a prospective study with participants recruited from the Cataract Department of the Hospital Oftalmológico de Brasília. Eligible subjects were aged 50 years or older, scheduled for bilateral phacoemulsification with implantation of the same IOL model, and had a best-corrected visual acuity of 20/50 or better. The IOL models included in the study were monofocal, premium monofocal, extended depth of focus (EDOF), and multifocal lenses. Patients with corneal diseases, retinal disorders, strabismus, or associated neuro-ophthalmological conditions were excluded. Only patients submitted to cataract surgery using the same type of IOL in both eyes were included in the study. The study procedures adhered to the Declaration of Helsinki for research involving human subjects and were approved by the Ethics Committee of the Hospital Oftalmológico de Brasília (IRB number 36566520.3.0000.5667). Informed consent was obtained from all participants (Tables 1, 2, 3, 4 and 5).

**Table 1:** Clinical and demographic characteristics of the patients included in the study

		IOL			
Characteristics	Total N = 58	EDOF N = 6	Monofocal/ Premium Monofocal N = 11	Trifocal N = 41	p-value
Participants					< 0.001^1^
	58 (100%)	6 (10.34%)	11 (18.97%)	41 (70.69%)	
Age					0.314^2^
Mean (SD)	64.53 (7.72)	69.67 (9.67)	66.36 (9.24)	63.29 (6.74)	
Median [Q1, Q3]	64.00 [60.00, 70.00]	68.50 [61.00, 74.00]	64.00 [62.00, 69.00]	63.00 [58.00, 69.00]	
Gender, n / N (%)					0.687^1^
Female	37 / 58 (63.79%)	3 / 6 (50.00%)	8 / 11 (72.73%)	26 / 41 (63.41%)	
Male	21 / 58 (36.21%)	3 / 6 (50.00%)	3 / 11 (27.27%)	15 / 41 (36.59%)	
Ethnicity, n / N (%)					0.549^1^
White	40 / 49 (81.63%)	3 / 4 (75.00%)	6 / 8 (75.00%)	31 / 37 (83.78%)	
Black/mixed race	9 / 49 (18.37%)	1 / 4 (25.00%)	2 / 8 (25.00%)	6 / 37 (16.22%)	
Education level, n / N (%)					0.076^1^
Elementary school complete	8 / 58 (13.79%)	1 / 6 (16.67%)	3 / 11 (27.27%)	4 / 41 (9.76%)	
Elementary school incomplete	3 / 58 (5.17%)	0 / 6 (0.00%)	2 / 11 (18.18%)	1 / 41 (2.44%)	
Higher education complete	47 / 58 (81.03%)	5 / 6 (83.33%)	6 / 11 (54.55%)	36 / 41 (87.80%)	
Occupational status, n / N (%)					0.424^1^
Retired	36 / 58 (62.07%)	5 / 6 (83.33%)	8 / 11 (72.73%)	23 / 41 (56.10%)	
Economically active	22 / 58 (37.93%)	1 / 6 (16.67%)	3 / 11 (27.27%)	18 / 41 (43.90%)	
History of refractive surgery, n / N (%)					0.153^1^
No	47 / 58 (81.03%)	4 / 6 (66.67%)	11 / 11 (100.00%)	32 / 41 (78.05%)	
Yes	11 / 58 (18.97%)	2 / 6 (33.33%)	0 / 11 (0.00%)	9 / 41 (21.95%)	
Mesopic pupillary diameter OD					0.439^2^
Mean (SD)	4.75 (1.29)	4.56 (1.04)	4.51 (0.90)	4.84 (1.41)	
Median [Q1, Q3]	4.92 [4.14, 5.52]	4.53 [3.86, 5.43]	4.54 [4.12, 5.29]	5.11 [4.15, 5.59]	
Mesopic pupillary diameter OS					0.186^2^
Mean (SD)	4.83 (0.99)	4.52 (0.98)	4.46 (1.00)	4.98 (0.98)	
Median [Q1, Q3]	4.61 [4.06, 5.68]	4.17 [3.90, 5.68]	4.42 [3.61, 5.46]	4.89 [4.17, 5.75]	
Photopic pupillary diameter OD					0.354^2^
Mean (SD)	3.48 (0.84)	3.44 (1.25)	3.20 (0.66)	3.56 (0.82)	
Median [Q1, Q3]	3.43 [2.89, 3.91]	3.03 [2.66, 3.43]	3.41 [2.43, 3.70]	3.53 [2.96, 4.09]	
Photopic pupillary diameter OS					0.492^2^
Mean (SD)	3.52 (0.85)	3.56 (1.16)	3.25 (0.70)	3.59 (0.85)	
Median [Q1, Q3]	3.41 [2.92, 4.05]	2.99 [2.93, 3.97]	3.22 [2.78, 3.87]	3.55 [2.97, 4.10]	
Cataract LOCS III OD, n / N (%)					0.241^1^
Nuclear 1	20 / 58 (34.48%)	0 / 6 (0.00%)	3 / 11 (27.27%)	17 / 41 (41.46%)	
Nuclear 2	26 / 58 (44.83%)	5 / 6 (83.33%)	6 / 11 (54.55%)	15 / 41 (36.59%)	
Others	12 / 58 (20.69%)	1 / 6 (16.67%)	2 / 11 (18.18%)	9 / 41 (21.95%)	
Cataract LOCS III OS, n / N (%)					0.241^1^
Nuclear 1	19 / 58 (32.76%)	0 / 6 (0.00%)	3 / 11 (27.27%)	16 / 41 (39.02%)	
Nuclear 2	28 / 58 (48.28%)	5 / 6 (83.33%)	6 / 11 (54.55%)	17 / 41 (41.46%)	
Others	11 / 58 (18.96%)	1 / 6 (16.67%)	2 / 11 (18.18%)	8 / 41 (19.52%)	
Femtosecond laser–assisted surgery, n / N (%)					0.077^1^
No	39 / 58 (67.24%)	6 / 6 (100.00%)	9 / 11 (81.82%)	24 / 41 (58.54%)	
Yes	19 / 58 (32.76%)	0 / 6 (0.00%)	2 / 11 (18.18%)	17 / 41 (41.46%)	
ND:YAG laser capsulotomy, n / N (%)					0.742^1^
No	39 / 58 (67.24%)	5 / 6 (83.33%)	8 / 11 (72.73%)	26 / 41 (63.41%)	
Yes	19 / 58 (32.76%)	1 / 6 (16.67%)	3 / 11 (27.27%)	15 / 41 (36.59%)	
Postoperative dependence of spectacles, n / N (%)					< 0.001^1^
No	45 / 58 (77.59%)	3 / 6 (50.00%)	2 / 11 (18.18%)	40 / 41 (97.56%)	
Yes	13 / 58 (22.41%)	3 / 6 (50.00%)	9 / 11 (81.82%)	1 / 41 (2.44%)	

^1^Fisher’s exact test

^2^Kruskal-Wallis rank sum test

IOL – Intraocular lenses; EDOF – Extended depth of focus; OD – Right eye; OS – Left eye; SD – Standard deviation; ND:YAG—Neodymium-Yttrium–Aluminum-Garnet

**Table 2:** Clinical changes before and after cataract surgery

	Moment		
Characteristics	1. Before surgery N = 58	2. After surgery N = 58	p-value
UDVA			0.002^1^
Mean (SD)	6.61 (2.56)	7.79 (2.38)	
Median [Q1, Q3]	7.50 [5.00, 8.00]	8.00 [7.00, 10.00]	
UNVA			< 0.001^1^
Mean (SD)	4.43 (3.00)	7.10 (2.57)	
Median [Q1, Q3]	3.50 [2.00, 7.00]	8.00 [5.00, 9.00]	
QoV_Frequency			0.860^1^
Mean (SD)	25.60 (17.00)	27.43 (18.12)	
Median [Q1, Q3]	25.15 [10.86, 35.48]	24.21 [14.47, 36.16]	
QoV_Severity			0.938^1^
Mean (SD)	26.62 (18.95)	27.24 (18.51)	
Median [Q1, Q3]	23.50 [13.47, 36.72]	23.46 [13.59, 36.76]	
QoV_Bothersome			0.669^1^
Mean (SD)	30.23 (23.27)	29.74 (23.66)	
Median [Q1, Q3]	25.66 [14.83, 38.11]	22.65 [11.01, 41.02]	
UDVA OD (logMAR)			< 0.001^1^
Mean (SD)	0.48 (0.37)	0.06 (0.11)	
Median [Q1, Q3]	0.40 [0.10, 0.70]	0.00 [0.00, 0.10]	
UDVA OS (logMAR)			< 0.001^1^
Mean (SD)	0.47 (0.34)	0.08 (0.11)	
Median [Q1, Q3]	0.40 [0.20, 0.70]	0.00 [0.00, 0.10]	
Spherical equivalent OD			0.016^1^
Mean (SD)	0.41 (2.29)	−0.09 (0.35)	
Median [Q1, Q3]	0.50 [−0.25, 1.75]	0.00 [−0.25, 0.00]	
Spherical equivalent OS			0.008^1^
Mean (SD)	0.46 (2.28)	−0.10 (0.35)	
Median [Q1, Q3]	0.75 [−0.50, 1.75]	0.00 [−0.25, 0.00]	

^1^Wilcoxon signed rank test with continuity correction

UDVA – Uncorrected distance visual acuity; UNVA – Uncorrected near visual acuity; OD – Right eye; OS – Left eye; QoV – Quality of vision questionnaire

**Table 3:** Clinical changes before and after cataract surgery in different types of intraocular lenses

	EDOF, N = 6			Monofocal/Premium Monofocal, N = 11			Trifocal, N = 41		
Characteristics	1. Before surgery CX N = 6	2. After surgery N = 6	p-value	1. Before surgery N = 11	2. After surgery N = 11	p-value	1. Before surgery N = 41	2. After surgery N = 41	p-value
UDVA			> 0.999			0.105			0.011
Mean (SD)	7.80 (2.28)	8.50 (1.52)		7.55 (1.81)	8.55 (1.29)		6.20 (2.69)	7.49 (2.66)	
Median [Q1; Q3]	8.00 [8.00; 9.00]	8.50 [8.00; 10.00]		7.00 [6.00; 9.00]	9.00 [8.00; 10.00]		7.00 [4.00; 8.00]	8.00 [7.00; 10.00]	
UNVA			0.197			0.120			< 0.001
Mean (SD)	5.20 (2.39)	6.50 (2.88)		3.45 (2.38)	5.36 (2.73)		4.60 (3.20)	7.66 (2.31)	
Median [Q1; Q3]	5.00 [4.00; 7.00]	6.50 [5.00; 9.00]		3.00 [1.00; 6.00]	5.00 [3.00; 8.00]		3.50 [1.50; 7.50]	8.00 [7.00; 9.00]	
QoV_Frequency			> 0.999			0.047			0.160
Mean (SD)	22.72 (18.19)	18.53 (13.86)		35.27 (21.29)	24.28 (17.83)		23.42 (15.01)	29.58 (18.57)	
Median [Q1; Q3]	24.81 [6.90; 28.25]	15.69 [10.51; 31.87]		35.48 [18.09; 51.87]	21.36 [10.86; 31.87]		21.36 [10.86; 34.46]	24.64 [17.75; 38.09]	
QoV_Severity			> 0.999			0.019			0.221
Mean (SD)	22.94 (19.88)	18.47 (13.54)		37.23 (24.19)	23.26 (18.56)		24.31 (16.66)	29.60 (18.91)	
Median [Q1; Q3]	23.61 [6.68; 26.95]	16.81 [10.08; 30.35]		33.87 [16.99; 60.24]	16.99 [10.19; 40.00]		23.44 [13.47; 33.98]	26.84 [16.87; 43.71]	
QoV_Bothersome			> 0.999			0.083			0.638
Mean (SD)	28.66 (26.78)	19.83 (16.55)		41.72 (29.87)	29.18 (29.06)		27.38 (20.34)	31.35 (23.12)	
Median [Q1; Q3]	26.06 [9.98; 32.69]	16.73 [7.60; 36.90]		34.30 [19.04; 65.88]	19.04 [7.60; 39.99]		25.46 [14.83; 35.47]	27.61 [15.23; 42.46]	
UDVA OD (logMAR)			0.098			0.004			< 0.001
Mean (SD)	0.37 (0.37)	0.10 (0.13)		0.69 (0.35)	0.11 (0.15)		0.44 (0.36)	0.04 (0.09)	
Median [Q1; Q3]	0.35 [0.00; 0.50]	0.05 [0.00; 0.20]		0.70 [0.30; 1.00]	0.10 [0.00; 0.20]		0.40 [0.10; 0.60]	0.00 [0.00; 0.00]	
UDVA OS (logMAR)			0.136			0.006			< 0.001
Mean (SD)	0.30 (0.22)	0.14 (0.10)		0.68 (0.36)	0.10 (0.14)		0.44 (0.33)	0.06 (0.10)	
Median [Q1; Q3]	0.25 [0.20; 0.50]	0.13 [0.10; 0.20]		0.70 [0.30; 0.90]	0.00 [0.00; 0.20]		0.40 [0.20; 0.70]	0.00 [0.00; 0.10]	
Spherical equivalent OD			0.786			0.343			0.016
Mean (SD)	−0.58 (1.38)	−0.08 (0.34)		0.89 (3.58)	−0.27 (0.51)		0.43 (1.95)	−0.04 (0.28)	
Median [Q1; Q3]	0.13 [−1.50; 0.25]	-0.13 [−0.25; 0.00]		0.50 [−0.75; 2.75]	−0.25 [−0.75; 0.00]		0.50 [0.00; 1.75]	0.00 [0.00; 0.00]	
Spherical equivalent OS			> 0.999			0.533			0.005
Mean (SD)	−0.42 (0.79)	−0.38 (0.38)		0.36 (3.98)	−0.20 (0.44)		0.62 (1.80)	−0.03 (0.30)	
Median [Q1; Q3]	−0.38 [−1.00; 0.25]	−0.38 [−0.50; 0.00]		0.75 [−2.25; 2.75]	0.00 [−0.25; 0.00]		1.00 [0.00; 1.75]	0.00 [0.00; 0.00]	

^1^Wilcoxon signed rank test with continuity correction

EDOF – Extended depth of focus; UDVA – Uncorrected distance visual acuity; UNVA – Uncorrected near visual acuity; SD – Standard deviation; OD – Right eye; OS – Left Eye; QoV – Quality of vision questionnaire

**Table 4:** Comparison between different types of intraocular lenses before and after cataract surgery

	1. Before surgery, N = 58				2. After surgery, N = 58			
Characteristics	EDOF N = 6	Monofocal/Premium Monofocal N = 11	Trifocal N = 41	p-value	EDOF N = 6	Monofocal/Premium Monofocal N = 11	Trifocal N = 41	p-value
UDVA				0.213				0.524
Mean (SD)	7.80 (2.28)	7.55 (1.81)	6.20 (2.69)		8.50 (1.52)	8.55 (1.29)	7.49 (2.66)	
Median [Q1; Q3]	8.00 [8.00; 9.00]	7.00 [6.00; 9.00]	7.00 [4.00; 8.00]		8.50 [8.00; 10.00]	9.00 [8.00; 10.00]	8.00 [7.00; 10.00]	
UNVA				0.430				0.039
Mean (SD)	5.20 (2.39)	3.45 (2.38)	4.60 (3.20)		6.50 (2.88)	5.36 (2.73)	7.66 (2.31)	
Median [Q1; Q3]	5.00 [4.00; 7.00]	3.00 [1.00; 6.00]	3.50 [1.50; 7.50]		6.50 [5.00; 9.00]	5.00 [3.00; 8.00]	8.00 [7.00; 9.00]	
QoV_Frequency				0.178				0.282
Mean (SD)	22.72 (18.19)	35.27 (21.29)	23.42 (15.01)		18.53 (13.86)	24.28 (17.83)	29.58 (18.57)	
Median [Q1; Q3]	24.81 [6.90; 28.25]	35.48 [18.09; 51.87]	21.36 [10.86; 34.46]		15.69 [10.51; 31.87]	21.36 [10.86; 31.87]	24.64 [17.75; 38.09]	
QoV_Severity				0.202				0.305
Mean (SD)	22.94 (19.88)	37.23 (24.19)	24.31 (16.66)		18.47 (13.54)	23.26 (18.56)	29.60 (18.91)	
Median [Q1; Q3]	23.61 [6.68; 26.95]	33.87 [16.99; 60.24]	23.44 [13.47; 33.98]		16.81 [10.08; 30.35]	16.99 [10.19; 40.00]	26.84 [16.87; 43.71]	
QoV_Bothersome				0.318				0.466
Mean (SD)	28.66 (26.78)	41.72 (29.87)	27.38 (20.34)		19.83 (16.55)	29.18 (29.06)	31.35 (23.12)	
Median [Q1; Q3]	26.06 [9.98; 32.69]	34.30 [19.04; 65.88]	25.46 [14.83; 35.47]		16.73 [7.60; 36.90]	19.04 [7.60; 39.99]	27.61 [15.23; 42.46]	
UDVA OD (logMAR)				0.074				0.069
Mean (SD)	0.37 (0.37)	0.69 (0.35)	0.44 (0.36)		0.10 (0.13)	0.11 (0.15)	0.04 (0.09)	
Median [Q1; Q3]	0.35 [0.00; 0.50]	0.70 [0.30; 1.00]	0.40 [0.10; 0.60]		0.05 [0.00; 0.20]	0.10 [0.00; 0.20]	0.00 [0.00; 0.00]	
UDVA OS (logMAR)				0.058				0.113
Mean (SD)	0.30 (0.22)	0.68 (0.36)	0.44 (0.33)		0.14 (0.10)	0.10 (0.14)	0.06 (0.10)	
Median [Q1; Q3]	0.25 [0.20; 0.50]	0.70 [0.30; 0.90]	0.40 [0.20; 0.70]		0.13 [0.10; 0.20]	0.00 [0.00; 0.20]	0.00 [0.00; 0.10]	
Spherical equivalent OD				0.181				0.250
Mean (SD)	−0.58 (1.38)	0.89 (3.58)	0.43 (1.95)		−0.08 (0.34)	−0.27 (0.51)	−0.04 (0.28)	
Median [Q1; Q3]	0.13 [−1.50; 0.25]	0.50 [−0.75; 2.75]	0.50 [0.00; 1.75]		−0.13 [−0.25; 0.00]	-0.25 [−0.75; 0.00]	0.00 [0.00; 0.00]	
Spherical equivalent OS				0.119				0.051
Mean (SD)	−0.42 (0.79)	0.36 (3.98)	0.62 (1.80)		−0.38 (0.38)	−0.20 (0.44)	−0.03 (0.30)	
Median [Q1; Q3]	−0.38 [−1.00; 0.25]	0.75 [−2.25; 2.75]	1.00 [0.00; 1.75]		−0.38 [−0.50; 0.00]	0.00 [−0.25; 0.00]	0.00 [0.00; 0.00]	

^1^Teste de Kruskal–Wallis

EDOF – Extended depth of focus; UDVA – Uncorrected distance visual acuity; UNVA – Uncorrected near visual acuity; SD – Standard deviation; OD – Right eye; OS – Left Eye; QoV – Quality of vision questionnaire

**Table 5:** Linear Mixed-Effects Models comparing Quality of Vision domains of Frequency, Severity and Bothersome in Different Intraocular Lenses

Characteristics	Regression coefficient (CI 95%)	p-value
Frequency Intraocular lenses		
EDOF	—	
Monofocal/ Premium Monofocal	12.6 (−4.93; 30.0)	0.157
Trifocal	0.70 (−14.4; 15.8)	0.926
Moment		
1. Before surgery	—	
2. After surgery	−4.19 (−20.1; 11.8)	0.601
Intraocular lenses * Moment		
Monofocal/ Premium Monofocal * 2. After surgery	−6.80 (−26.6; 13.0)	0.495
Trifocal * 2. After surgery	10.3 (−6.74; 27.4)	0.230
Severity Intraocular lenses		
EDOF	—	
Monofocal/ Premium Monofocal	14.3 (−4.33; 32.9)	0.131
Trifocal	1.37 (−14.7; 17.4)	0.866
Moment		
1. Before surgery	—	
2. After surgery	−4.47 (−21.6; 12.7)	0.604
Intraocular lenses * Moment		
Monofocal/ Premium Monofocal * 2. After surgery	−9.50 (−30.9; 11.8)	0.376
Trifocal * 2. After surgery	9.76 (−8.63; 28.1)	0.292
Bothersome Intraocular lenses		
EDOF	—	
Monofocal/ Premium Monofocal	13.1 (−10.5; 36.6)	0.275
Trifocal	−1.29 (−21.6; 19.0)	0.900
Moment		
1. Before surgery	—	
2. After surgery	−8.84 (−29.0; 11.3)	0.384
Intraocular lenses * Moment		
Monofocal/ Premium Monofocal * 2. After surgery	−3.70 (−28.8; 21.4)	0.769
Trifocal * 2. After surgery	12.8 (−8.80; 34.4)	0.240

EDOF – Extended depth of focus; CI – Confidence interval

### Pre- and postoperative follow-up

In the preoperative evaluation, patients underwent a comprehensive ophthalmologic examination, including measurement of visual acuity with and without correction using electronic ETDRS optotypes and quantification in logMAR, slit-lamp biomicroscopy, and cataract grading according to the Lens Opacities Classification System III (LOCS III) [10]. Additional assessments included Goldmann applanation tonometry, retinal mapping, specular microscopy (CEM-530, Nidek – Japan), aberrometry (OPD-Scan III, Nidek – Japan), corneal tomography (Pentacam, Oculus – Germany), and optical biometry (IOLMaster 500, Zeiss – Germany). Postoperative evaluations were performed on the first postoperative day, including uncorrected visual acuity measurement, slit-lamp biomicroscopy, and fundoscopic examination, and on the 30th postoperative day, when all tests were repeated except for biometry. The Quality of Vision questionnaire (Table 6) was completed after the initial preoperative assessment and again at the 30-day postoperative evaluation. A relatively short follow-up period of 30 days was adopted, which may limit the assessment of long-term visual quality outcomes and neuroadaptation processes.

**Table 6:** Quality of Vision (QoV) Questionnaire translated into Brazilian Portuguese

Questions
Com que frequência você tem ofuscamento na sua visão? [1]
Qual a intensidade deste ofuscamento? [2]
O quanto este ofuscamento te incomoda? [3]
Com que frequência você apresenta halos na visão? [1]
Qual a intensidade dos halos? [2]
O quanto os halos te incomodam? [3]
Com que frequência você apresenta “explosão de estrelas” na visão? [1]
Qual a intensidade dessa “explosão de estrelas”? [2]
O quanto a “explosão de estrelas” te incomoda? [3]
Com que frequência você apresenta visão enevoada? [1]
Qual a intensidade dessa visão enevoada? [2]
O quanto a visão enevoada te incomoda? [3]
Com que frequência você apresenta visão borrada? [1]
Qual a intensidade dessa visão borrada? [2]
O quanto essa visão borrada te incomoda? [3]
Com que frequência você apresenta visão distorcida? [1]
Qual a intensidade dessa visão distorcida? [2]
O quanto essa visão distorcida te incomoda? [3]
Com que frequência você apresenta visão dupla? [1]
Qual a intensidade dessa visão dupla? [2]
O quanto essa visão dupla te incomoda? [3]
Com que frequência você apresenta flutuação da visão? [1]
Qual a intensidade da flutuação da visão? [2]
O quanto a flutuação na visão te incomoda? [3]
Com que frequência você apresenta dificuldade para focar a visão? [1]
Qual a intensidade dessa dificuldade para focar a visão? [2]
O quanto a dificuldade para focar a visão te incomoda? [3]
Com que frequência você apresenta dificuldade para enxergar profundidade e distância? [1]
Qual a intensidade dessa dificuldade para enxergar profundidade e distância? [2]
O quanto essa dificuldade para enxergar profundidade e distância te incomoda? [3]
Possible response options for frequency questions [1]
Nunca
Ocasionalmente
Frequentemente
Muito frequentemente
Possible response options for severity questions [2]
Nenhuma
Leve
Moderada
Grave
Possible response options for bothersome questions [3]
Nada
Um pouco
Moderado
Muito

Patients who developed clinically significant posterior capsule opacification during postoperative follow-up underwent Nd laser capsulotomy before completion of the final QoV assessment. In these cases, the QoV questionnaire was administered only after visual recovery following the laser procedure, ensuring that questionnaire responses reflected the patient’s final postoperative visual status.

### Surgery

All patients were operated on by the same experienced surgeon under topical anesthesia combined with sedation. The main incision was made along the steepest meridian to minimize postoperative induced astigmatism. Surgeries were performed using the Proveo 8X surgical microscope (Leica – Germany) and the Centurion phacoemulsification system (Alcon – United States). No intraoperative complications occurred, and all intraocular lenses were implanted within the capsular bag. At the end of the procedure, all patients received an intracameral injection of 0.2 mL of moxifloxacin (5.45 mg/mL). Postoperatively, patients were prescribed topical moxifloxacin 5 mg/mL + dexamethasone 1 mg/mL for 7 days, followed by prednisolone 10 mg/mL in a tapering regimen from the second to the fifth week, and ketorolac tromethamine 5 mg/mL for 5 weeks. The postoperative medication regimen was standardized for all patients. Surgeries were performed with an interval of 48 h between eyes.

### Intraocular lenses

Monofocal IOLs, typically made of hydrophobic or hydrophilic acrylic and designed as single-piece optics, feature either spherical or aspheric profiles and provide a single focal point for distance vision, ensuring high optical quality and contrast [4]. In this study, the Sensar AAB00 (Johnson & Johnson) and AcrySof SA60AT (Alcon) lenses were used. Premium monofocal IOLs incorporate design enhancements such as negative spherical aberration, continuous square-edge profiles, and light filters, offering greater depth of focus and reduced dysphotopsia compared with conventional monofocal lenses [11]. The lens used in this study was the Tecnis Eyhance (Johnson & Johnson). For analytical purposes, premium monofocal IOLs were grouped together with conventional monofocal lenses, as they share a single focal point and similar visual performance profiles, despite minor design enhancements such as improved depth of focus. EDOF IOLs employ refractive, diffractive, or hybrid optical designs that extend the range of vision without creating distinct focal points, improving intermediate vision while maintaining good contrast sensitivity [12]. The Tecnis Symfony (Johnson & Johnson) and AcrySof IQ Vivity (Alcon) lenses were used in this group. Trifocal IOLs, mostly composed of hydrophilic acrylic material with diffractive surfaces, distribute light across three principal foci—distance, intermediate, and near—providing spectacle independence at multiple distances. The light distribution is typically around 40–45% for distance, 15–20% for intermediate, and 25–30% for near vision [13]. The trifocal lenses used in this study were PanOptix and Clareon PanOptix (Alcon), Vivinex Gemetric (Hoya), Intensity SL (Hanita), Tecnis MIOL, and Tecnis Synergy (Johnson & Johnson).

### Quality of vision (QoV) questionnaire and translation into portuguese

The QoV questionnaire consists of 10 items related to visual symptoms, each evaluated across three domains: frequency, severity, and bothersome [14]. The visual symptoms assessed include glare, halos, starbursts, hazy vision, blurred vision, distortion, fluctuation, focusing difficulties, double vision, and difficulty in judging distances. Developed using Rasch analysis, each of its 30 items offers four response options to discriminate symptom intensity and is written at a 12-year-old reading level [2, 6].

The Quality of Vision (QoV) questionnaire was translated into Brazilian Portuguese following standardized procedures for cross-cultural adaptation, as described by Beaton and Gjersing (Table 6) [15–17]. Initially, two independent translations of the original English version were performed—one by a cataract specialist ophthalmologist and another by a general ophthalmologist, both fluent in English and Brazilian Portuguese. A third reviewer, fluent in both languages, revised, reconciled, and synthesized the two versions into a single unified version, with the agreement of the original translators. Subsequently, the synthesized version was back translated into English by two different professionals: a cataract surgeon and a certified translator who was a native English speaker residing in Brazil. The same reviewer responsible for synthesizing the translations evaluated the back-translations and produced a consolidated version. Differences in terminology were discussed and resolved by consensus before inclusion in the final version. Finally, an expert committee—composed of ophthalmologists and the certified translator—compared, item by item, the original English version, the Portuguese translation, and the back-translated English version. The committee made targeted adjustments to ensure clarity, conceptual equivalence, and content fidelity.

The translation process followed internationally accepted guidelines for cross-cultural adaptation and sought to ensure semantic, idiomatic, experiential, and conceptual equivalence between the original and Brazilian Portuguese versions [16, 17]. The absence of major discrepancies during forward translation, back-translation, and expert committee review supports the cross-cultural equivalence of the final instrument.

### Statistical analysis

Descriptive statistics (mean, median, standard deviation, interquartile range, absolute frequencies, and percentages) were used to summarize the data. Associations between categorical variables were tested using the Chi-square test, and Fisher’s exact test was applied when the sample size was small [18]. Data normality was assessed with the Shapiro–Wilk test, which indicated a non-normal distribution. For nonparametric comparisons, the Mann–Whitney U test was used for independent groups, and the Wilcoxon signed-rank test was applied for paired samples. When comparing three or more groups, the Kruskal–Wallis test (for independent samples) and the Friedman test (for paired samples) were employed. When the global nonparametric tests were significant, post-hoc analyses were performed using Dunn’s test after Kruskal–Wallis and Nemenyi’s test after Friedman [19]. Linear Mixed-Effects Models (LMMs) were used to analyze hierarchical and repeated-measures data, incorporating both fixed and random effects to account for intra- and intergroup variability [20]. These models were considered the primary analytical approach for comparing QoV outcomes between IOL groups over time, as they appropriately handle within-subject correlations and repeated observations. All analyses were performed using the R statistical environment (version 4.3.2), with the level of significance set at 5% (p < 0.05).

### Rasch analysis

Rasch analysis was performed and demonstrated acceptable psychometric performance of the Portuguese QoV questionnaire. Person separation index values ranged from 1.78 to 1.87, with reliability coefficients between 0.77 and 0.78 across the frequency, severity, and bothersome domains, indicating borderline but acceptable discriminative ability for group-level comparisons [21]. The relatively modest person separation values suggest that the questionnaire may be more suitable for group-level analyses than for detecting subtle differences between individual patients. A detailed description of the Rasch methodology and full validation results are provided in the Supplemental Material.

## Results

A total of 58 patients were included in the study, of whom 10.34% belonged to the EDOF lens group, 18.97% to the monofocal IOL group, and 70.69% to the trifocal lens group (*p* < 0.001). Clinical and demographic characteristics of subjects included in the study are detailed in Table 1. Mean age was 64.53 years (*p* = 0.314); 63.79% of the patients were female and 36.21% male (*p* = 0.687). Regarding ethnicity, 81.63% were White and 18.37% were Black or mixed race (*p *= 0.549). As for education level, 13.79% had completed elementary school, 5.17% had incomplete elementary education, and 81.03% had completed higher education (*p* = 0.076). In terms of occupational status, 62.07% of the total sample were retired, while 37.93% were economically active (*p* = 0.424).

Among all participants, 81.03% reported no history of refractive surgery, compared to 18.97% who had undergone such procedures (*p* = 0.153). Cataract classification according to the Lens Opacities Classification System III (LOCS III) showed a predominance of N1 (34.48%) and N2 (44.83%) cataracts in the right eye (*p* = 0.241), and N1 (32.76%) and N2 (48.28%) in the left eye (*p* = 0.241). Approximately 67.24% of patients underwent femtosecond laser–assisted surgery, while 32.76% did not (*p* = 0.077). Neodymium-Yttrium–Aluminum-Garnet (Nd) laser capsulotomy was performed in 32.76% of patients who developed clinically significant posterior capsule opacification during postoperative follow-up. In these cases, the final QoV questionnaire was administered only after recovery from the laser procedure. The frequency of Nd capsulotomy did not differ significantly among IOL groups (*p* = 0.742) (Table 1). Postoperative dependence on spectacles was observed in 22.41% of all patients, distributed as 81.82% in the monofocal group, 50% in the EDOF group, and only 2.44% in the trifocal group (*p* < 0.001) (Table 1).

In the total cohort, the median satisfaction score for uncorrected distance visual acuity (UDVA) was 8.00 after surgery compared to 7.50 before surgery (*p* = 0.002), while median satisfaction for near vision improved from 3.50 before surgery to 8.00 postoperatively (*p* < 0.001) (Table 2). The median uncorrected visual acuity (logMAR) for both right and left eyes improved from 0.40 before surgery to 0.00 after surgery (*p* < 0.001). The median spherical equivalent for the right eye decreased from + 0.50 before surgery to 0.00 postoperatively (*p* = 0.016), and for the left eye from + 0.75 before surgery to 0.00 after surgery (*p* = 0.008) (Table 2). UDVA satisfaction in the trifocal lens group showed a median of 8.00 after surgery compared to 7.00 before surgery (*p* = 0.011), while near vision satisfaction improved from a median of 3.50 before surgery to 8.0 after surgery in the same group (*p* < 0.001) (Table 2).

**Fig. 1:**
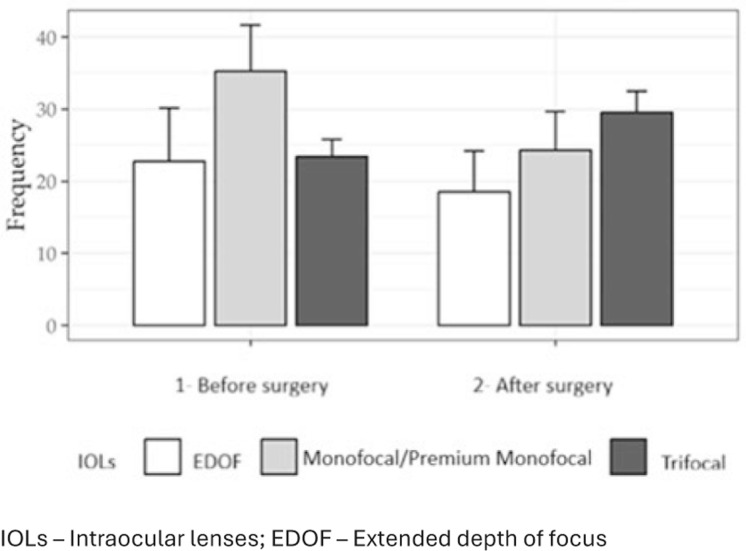
Quality of Vision Questionnaire Results evaluating Frequency before and after cataract surgery comparing different intraocular lenses. IOLs – Intraocular lenses; EDOF – Extended depth of focus

**Fig. 2:**
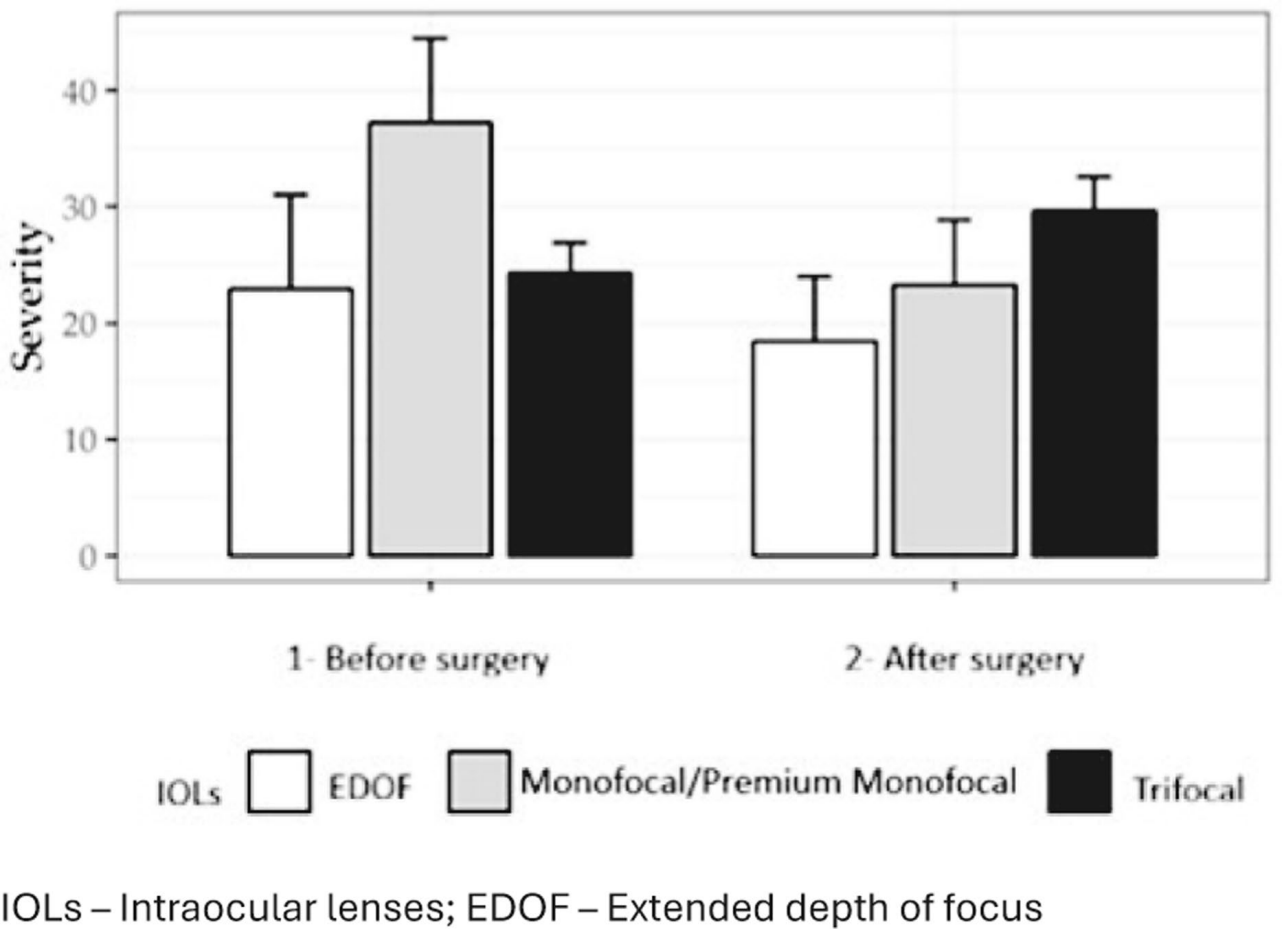
Quality of Vision Questionnaire Results evaluating Severity before and after cataract surgery comparing different intraocular lenses. IOLs – Intraocular lenses; EDOF – Extended depth of focus

**Fig. 3:**
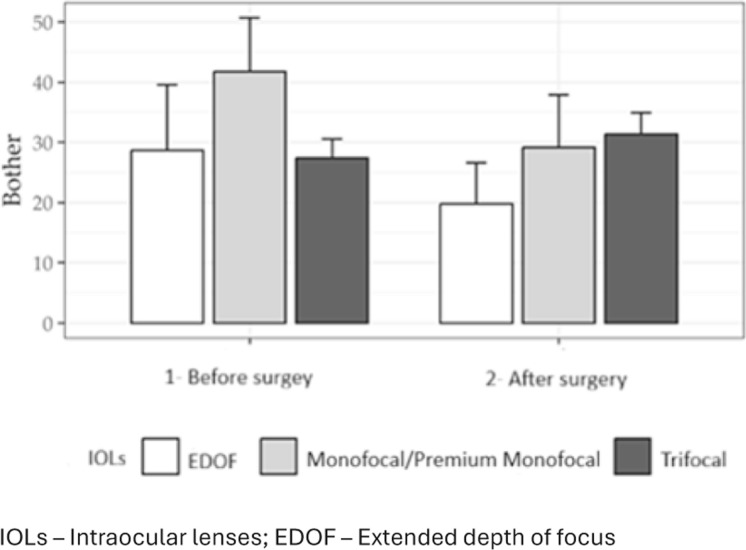
Quality of Vision Questionnaire Results evaluating Bothersome before and after cataract surgery comparing different intraocular lenses. IOLs – Intraocular lenses; EDOF – Extended depth of focus

We compared QoV domains in the 3 different IOL groups before and after cataract surgery in Tables 3 and 4 and Figs. 1, 2 and 3. The QoV frequency domain showed a median of 21.36 after surgery versus 35.48 before surgery in the monofocal lens group (*p* = 0.047). The QoV severity domain showed a median of 16.99 after surgery versus 33.87 before surgery in the same group (*p* = 0.019). For the right eye (OD), the UDVA median in the monofocal lens group was 0.10 after surgery versus 0.70 before surgery (*p* = 0.04), whereas in the trifocal group it was 0.00 after surgery versus 0.40 before surgery (*p* < 0.001).

For the left eye (OS), the UDVA median in the monofocal group was 0.00 after surgery versus 0.70 before surgery (*p* = 0.006), while in the trifocal group it was 0.00 after surgery versus 0.40 before surgery (*p* < 0.001). The spherical equivalent for the right eye in the trifocal lens group showed a median of 0.00 after surgery versus 0.50 before surgery (*p* = 0.016), while for the left eye in the same group, the median was 0.00 after surgery versus 1.00 before surgery (*p* = 0.005) (Table 3 and 4).

We also performed a LMM analysis to compare the QoV outcomes (frequency, severity and bothersome) between the 3 different IOL types, before and after cataract surgery and the interaction between the variables (Table 5), but none of the parameters showed statistical differences.

## Discussion

As different types of IOL are constantly developed, it is crucial to investigate the impact on the QoV of patients submitted to cataract surgery. Thus, we performed a longitudinal study comparing subjective complaints before and after cataract surgery using different IOL platforms and a validated questionnaire. This is the first study in Brazil to use the Portuguese translated version of the QoV in a longitudinal study comparing outcomes before and after surgery.

The Quality of Vision Questionnaire was developed to measure the subjective perception of visual quality, including symptoms such as halos, glare, and optical distortions, which are often not detected by traditional visual acuity tests [2, 22]. The instrument demonstrated high internal consistency, reliability, and structural robustness, with the possibility of interchange between its subscales as it has been previously validated using modern psychometric methods [14]. The questionnaire has proven reliable in both printed and digital versions, the latter being more practical without loss of accuracy, and has been applied in the subjective evaluation of surgical outcomes and in comparisons between different intraocular lens models [23].

The results showed a significant improvement in UDVA in the postoperative period, with a median of 0.40 to 0.0 in both eyes (*p* < 0.001) (Table 2). This finding corroborates classical studies demonstrating the positive impact of cataract surgery on the restoration of functional vision, regardless of the type of implanted intraocular lens [3]. Regarding the global spherical equivalent, a statistically significant reduction was observed, indicating a trend toward postoperative emmetropization. The median decreased from 0.50 to 0.0 D in the right eye (*p* = 0.016) and from 0.75 to 0.0 D in the left eye (*p* = 0.008) (Table 2). These findings reinforce the refractive accuracy achieved by modern phacoemulsification techniques combined with optical biometry, aligning with previous investigations that demonstrate the consistency of refractive outcomes across different instruments and IOL calculation protocols [24].

Despite these objective improvements, QoV scores did not show statistically significant differences between the preoperative and postoperative periods for the frequency (25.15 vs. 24.21; p = 0.860), severity (23.50 vs. 23.46; *p* = 0.938), and bothersome (25.66 vs. 22.56; *p* = 0.669) domains (Table 2). This apparent discrepancy may be explained by the baseline characteristics of the sample. Most patients presented with mild cataracts, predominantly classified as LOCS III [10], with 79.31% of right eyes and 81.04% of left eyes classified as N1 or N2 (Table 1), suggesting low preoperative symptomatology and, consequently, a lower potential for noticeable improvement in dysphotopsia symptoms. In addition, a possible ceiling effect in patient-reported outcome measures (PROMs) should be considered, as relatively high baseline QoV scores may have reduced sensitivity to postoperative changes. Furthermore, many participants already reported satisfactory visual quality before surgery, leaving limited room for measurable improvement in QoV outcomes despite substantial gains in visual acuity and patient satisfaction. This discrepancy may also reflect the distinct constructs measured by these outcomes. While visual acuity and satisfaction primarily assess functional performance and patient expectations, the QoV questionnaire focuses on specific visual symptoms such as glare, halos, starbursts, and other dysphotopsias [2]. Consequently, significant improvements in objective visual function may occur without corresponding changes in symptom-based QoV scores. These factors should be taken into account when interpreting the stability of QoV outcomes observed in this study. These results are consistent with studies that also did not identify a significant increase in photopic symptoms after implantation of different IOL models [24, 25]. Furthermore, psychosocial factors and personality traits, such as neuroticism and introversion, have been shown to influence the subjective perception of vision and, therefore, QoV scores [26].

Post hoc analyses demonstrated predominantly low statistical power across QoV outcomes despite moderate effect sizes in selected comparisons, particularly within the monofocal group. Cohen’s d values suggested clinically meaningful improvements in QoV frequency and severity among monofocal IOL recipients, whereas trifocal IOLs demonstrated small effect sizes, consistent with a trend toward increased dysphotopsia. Although significant within-group improvements were observed in selected monofocal comparisons, these findings were not confirmed in the linear mixed-effects models. Because mixed-effects models account for repeated measurements, between-group variability, and time-by-group interactions, they should be considered the primary inferential analysis in this study. Notably, none of the interaction terms between time and IOL type reached statistical significance, and most comparisons were associated with low statistical power. Therefore, the absence of significant between-group differences should not be interpreted as evidence of equivalence among IOL categories, but rather as a finding that may reflect the limited ability of the study to detect subtle yet clinically meaningful differences. Detailed post hoc power analyses, effect sizes, and Cohen’s d values are provided in the Supplemental Material (Tables 7 and 8).

In the comparison between different types of IOLs, trifocal lenses showed the highest rate of postoperative spectacle independence, with only 2.44% of patients requiring additional correction after surgery (*p* < 0.001) (Table 1), a result widely confirmed in the literature [26, 27]. However, the overall QoV questionnaire scores, considering the frequency, severity, and bothersome domains, did not show statistically significant differences between the groups with trifocal, EDOF, and monofocal IOLs (Table 4). Although a trend toward worsening dysphotopsia symptoms was observed among patients with trifocal IOLs, reflected by a slight increase in the medians after surgery in the items: frequency from 21.36 to 24.64 (*p* = 0.160), severity from 23.44 to 26.84 (*p* = 0.221), and bothersome from 25.46 to 27.61 (*p* = 0.683) (Table 3). Despite the lack of statistical significance, these results may suggest a trend toward greater perception of photic phenomena in trifocal IOLs, possibly related to the multiple-diffractive design of these lenses [25].

From a clinical perspective, the absence of substantial deterioration in QoV among trifocal and EDOF recipients despite greater spectacle independence may be reassuring for surgeons counseling patients regarding premium IOL implantation. These findings suggest that improvements in visual function can be achieved without a measurable increase in subjective visual disturbances during the early postoperative period [24, 25].

## Limitations

The current study has several important limitations. First, the relatively small sample size reduced the statistical power to detect subtle differences between IOL categories, increasing the risk of Type II error. This limitation was further compounded by the marked imbalance among groups, with a predominance of trifocal IOL recipients and comparatively few patients in the EDOF and monofocal groups, which may have influenced comparative analyses. Although no significant differences were observed between groups regarding age, sex, ethnicity, education level, or pupillary diameter (Table 1), suggesting a degree of baseline homogeneity, the high proportion of trifocal lens recipients, the predominance of highly educated participants, and recruitment from a single private ophthalmology center may limit the generalizability of these findings to broader cataract populations.

Second, incomplete questionnaire responses and losses to follow-up reduced the final sample available for longitudinal analyses. In addition, the 30-day follow-up period may have been insufficient to fully evaluate neuroadaptation, particularly among recipients of premium IOLs. Adaptation to photic phenomena such as halos and glare may continue for several months after surgery, and longer follow-up periods could reveal changes in dysphotopsia symptoms and subjective visual quality that were not captured in the present study. An additional consideration is that approximately one-third of the patients required Nd laser capsulotomy for posterior capsule opacification before the final QoV assessment. Although the questionnaire was administered only after recovery from the procedure, the potential influence of posterior capsule opacification and subsequent laser treatment on subjective visual quality cannot be completely excluded.

Third, although Rasch validation demonstrated acceptable overall psychometric performance of the Portuguese version of the QoV questionnaire, the person separation indices (1.78–1.87) and person reliability coefficients (0.77–0.78) were slightly below the thresholds typically considered desirable for robust discrimination among individuals. These findings suggest that the instrument was capable of distinguishing broad levels of visual quality but may have had limited sensitivity to detect subtle differences between patients with similar visual experiences or between closely related IOL categories. Therefore, the absence of significant differences in QoV outcomes between IOL groups should be interpreted with caution, as small but clinically meaningful differences may not have been fully captured by the questionnaire. Future studies with larger samples and additional psychometric evaluation may help further refine the measurement properties of the Brazilian Portuguese QoV instrument.

## Conclusion

In summary, cataract surgery significantly improved visual acuity and subjective visual satisfaction across all IOL groups. However, no statistically significant differences in QoV outcomes were detected between IOL categories during the early postoperative period. Considering the small and highly unbalanced sample, these findings should not be interpreted as evidence of equivalence among IOL types, and larger studies are needed to determine whether clinically meaningful differences exist.

## Supplementary Information

Below is the link to the electronic supplementary material.

Supplementary file1 (XLSX 49 KB)

Supplementary file2 (DOCX 16 KB)

Supplementary file3 (DOCX 19 KB)

Supplementary file4 (DOCX 17 KB)

## Data Availability

No datasets were generated or analysed during the current study.
