# Supplementary material for: Assessment of the impact of cataract surgery on subjective quality of vision across different intraocular lens type using the portuguese-validated QoV questionnaire
Source: Int Ophthalmol. 2026 Jul 20;46(1):301. doi: 10.1007/s10792-026-04172-x (PMC13384983; doi:10.1007/s10792-026-04172-x)
Supplement: Supplementary file 2 — Supplementary file2 (DOCX 16 KB) [file 10792_2026_4172_MOESM2_ESM.docx]

**Rasch Analysis:**

Rasch analysis was used to position participants and QoV items on the same linear interval scale, expressed in logits (log-odds units), simultaneously estimating individual ability and item difficulty through the Andrich rating scale model [2, 21]. Model fit was assessed using infit and outfit mean-square (MNSQ) statistics, with acceptable values ranging between 0.7 and 1.3. Differential item functioning (DIF) was evaluated to detect potential differences among subgroups, considered absent when <0.5 logits, minimal between 0.5–1.0 logits, and significant when >1.0 logits. Measurement precision was determined using the person separation index (PSI) and person separation reliability, with acceptable thresholds defined as PSI ≥ 2.0 or reliability ≥ 0.8.

For the validation of the Portuguese version of the QoV questionnaire, an independent sample of 236 patients completed the instrument before and after cataract surgery. In our validation sample, PSI values were 1.78 (frequency), 1.85 (severity), and 1.87 (bothersome), with corresponding reliability coefficients of 0.78, 0.77, and 0.78, respectively. Although these values are slightly below the conventional thresholds, they still indicate acceptable, albeit borderline, discriminative ability of the instrument. Specifically, PSI values below 2.0 suggest a reduced capacity to distinguish between multiple distinct strata of visual quality perception, particularly among individuals with similar symptom levels. However, the reliability coefficients approaching 0.80 support the consistency of the measurements.

From a clinical perspective, these findings indicate that the Portuguese version of the QoV questionnaire is adequate for group-level comparisons and longitudinal assessment, as performed in the present study, but may have limited sensitivity for detecting small differences between closely related levels of subjective visual quality. Consequently, small differences between individual participants or between closely related IOL categories may not be fully captured by the instrument, particularly in studies with limited sample sizes.

All analyses were conducted using IBM SPSS version 21, Winsteps version 3.81.0, and Stata version 13.
