# Supplementary material for: Assessment of the impact of cataract surgery on subjective quality of vision across different intraocular lens type using the portuguese-validated QoV questionnaire
Source: Int Ophthalmol. 2026 Jul 20;46(1):301. doi: 10.1007/s10792-026-04172-x (PMC13384983; doi:10.1007/s10792-026-04172-x)
Supplement: Supplementary file 3 — Supplementary file3 (DOCX 19 KB) [file 10792_2026_4172_MOESM3_ESM.docx]

**Table 7: Post-hoc power calculation**

|  | **Before the surgery** | | | | | | **After the surgery** | | | | | |
| --- | --- | --- | --- | --- | --- | --- | --- | --- | --- | --- | --- | --- |
|  | **EDOF vs Mono** | | **EDOF vs Tri** | | **Mono vs Tri** | | **EDOF vs Mono** | | **EDOF vs Tri** | | **Mono vs Tri** | |
| **Variant** | **d** | **Pwr** | **d** | **Pwr** | **d** | **Pwr** | **d** | **Pwr** | **d** | **Pwr** | **d** | **Pwr** |
| UDVA | 0.126 | 0.056 | 0.604 | 0.272 | 0.532 | 0.336 | -0.036 | 0.051 | 0.395 | 0.143 | 0.433 | 0.240 |
| UNVA | 0.734 | 0.273 | 0.192 | 0.071 | -0.377 | 0.193 | 0.410 | 0.118 | -0.487 | 0.194 | -0.958 | 0.790 |
| QoV_F | -0.618 | 0.207 | -0.045 | 0.051 | 0.720 | 0.548 | -0.346 | 0.098 | -0.610 | 0.277 | -0.288 | 0.132 |
| QoV_S | -0.626 | 0.211 | -0.080 | 0.054 | 0.702 | 0.527 | -0.281 | 0.081 | -0.605 | 0.273 | -0.337 | 0.163 |
| QoV_B | -0.452 | 0.133 | 0.061 | 0.052 | 0.635 | 0.450 | -0.366 | 0.104 | -0.512 | 0.209 | -0.089 | 0.058 |
| UDVA_OD | -0.897 | 0.380 | -0.194 | 0.072 | 0.698 | 0.523 | -0.070 | 0.052 | 0.630 | 0.292 | 0.668 | 0.488 |
| UDVA_OE | -1.187 | 0.590 | -0.438 | 0.165 | 0.714 | 0.541 | 0.312 | 0.089 | 0.800 | 0.433 | 0.366 | 0.185 |
| SE_OD | -0.485 | 0.146 | -0.533 | 0.222 | 0.194 | 0.087 | 0.413 | 0.119 | -0.139 | 0.061 | -0.679 | 0.501 |
| SE_OE | -0.238 | 0.072 | -0.606 | 0.273 | -0.108 | 0.061 | -0.428 | 0.124 | -1.129 | 0.715 | -0.511 | 0.314 |

UDVA – Uncorrected distance visual acuity; UNVA – Uncorrected near visual acuity; OD – Right eye; OS – Left Eye; QoV – Quality of vision questionnaire; F – Frequency; S – Severity; B – Bothersome; SE – Spherical equivalent
