# Supplementary material for: Assessment of the impact of cataract surgery on subjective quality of vision across different intraocular lens type using the portuguese-validated QoV questionnaire
Source: Int Ophthalmol. 2026 Jul 20;46(1):301. doi: 10.1007/s10792-026-04172-x (PMC13384983; doi:10.1007/s10792-026-04172-x)
Supplement: Supplementary file 4 — Supplementary file4 (DOCX 17 KB) [file 10792_2026_4172_MOESM4_ESM.docx]

**Table 8: Cohen’s d effect sizes**

|  | **Cohen's d** | | |
| --- | --- | --- | --- |
| **Variável** | **EDOF** | **Monofocal** | **Trifocal** |
| UDVA | 0.361 (0.112) | 0.636 (0.479) | 0.482 (0.854) |
| UNVA | 0.491 (0.166) | 0.746 (0.607) | 1.096 (1.000) |
| QoV_Frequency | -0.259 (0.082) | -0.560 (0.389) | 0.365 (0.625) |
| QoV_Severity | -0.263 (0.083) | -0.648 (0.493) | 0.297 (0.458) |
| QoV_Bothersome | -0.397 (0.125) | -0.426 (0.248) | 0.182 (0.207) |
| UDVA_OD | -0.974 (0.487) | -2.154 (1.000) | -1.524 (1.000) |
| UDVA_OS | -0.936 (0.458) | -2.124 (1.000) | -1.559 (1.000) |
| SE_OD | 0.498 (0.169) | -0.454 (0.275) | -0.337 (0.559) |
| SE_OS | 0.065 (0.052) | -0.198 (0.091) | -0.504 (0.882) |
| Values shown as Cohen's d (statistical power 1-β).  UDVA – Uncorrected distance visual acuity; UNVA – Uncorrected near visual acuity; OD – Right eye; OS – Left Eye; QoV – Quality of vision questionnaire; F – Frequency; S – Severity; B – Bothersome; SE – Spherical equivalent | | | |
